# A modular *“Catch-and-Play”* platform for rapid T-cell engager target assembly for personalised cancer treatment

**DOI:** 10.1038/s41392-025-02557-5

**Published:** 2026-01-12

**Authors:** Xi Xi, Yonghui Zhang, Daqing Zhao, Fangfang Chen, Kenneth A. Howard

**Affiliations:** 1 Key Laboratory of Pathobiology, Ministry of Education, Nanomedicine and Translational Research Center, China-Japan Union Hospital of Jilin University, Jilin, China; 2 Interdisciplinary Nanoscience Center (iNANO) and Department of Molecular Biology and Genetics, Aarhus University, DK-8000 Aarhus C, Denmark; 3 Northeast Asia Research Institute of Traditional Chinese Medicine, Changchun University of Chinese Medicine, Jilin, China

**Keywords:** Protein delivery, Molecular engineering

**Dear editor**,

Bispecific T-cell engagers (also referred to as BiTEs) are bi/multi-specific antibodies that are a clinically used cancer immunotherapy, which enforce formation of an immunological synapse between T-cells and tumour cells by antigenic engagement on the respective cells, resulting in cancer cell apoptosis.^1,2^ Tumour heterogeneity, however, is a major challenge for identifying the most appropriate cancer target antigen for each patient. Discovery of new tumour targets outweighs new BiTE approval, calling for rapid screening tools to evaluate novel targets and BiTE combinational formats. Although several multifunctional BiTEs platforms have been explored, a simple, rapid, and safe platform needs to be developed. Here we introduce an albumin-based *“Catch-and-Play”* modular SpyCatcher/SpyTag conjugation platform for potential rapid assembly of interchangeable targeting modules personalised to the patient tumour antigenic profile, and an FcRn-driven half-life extension to reduce the required dose (Fig. 1a).

**Fig. 1:**
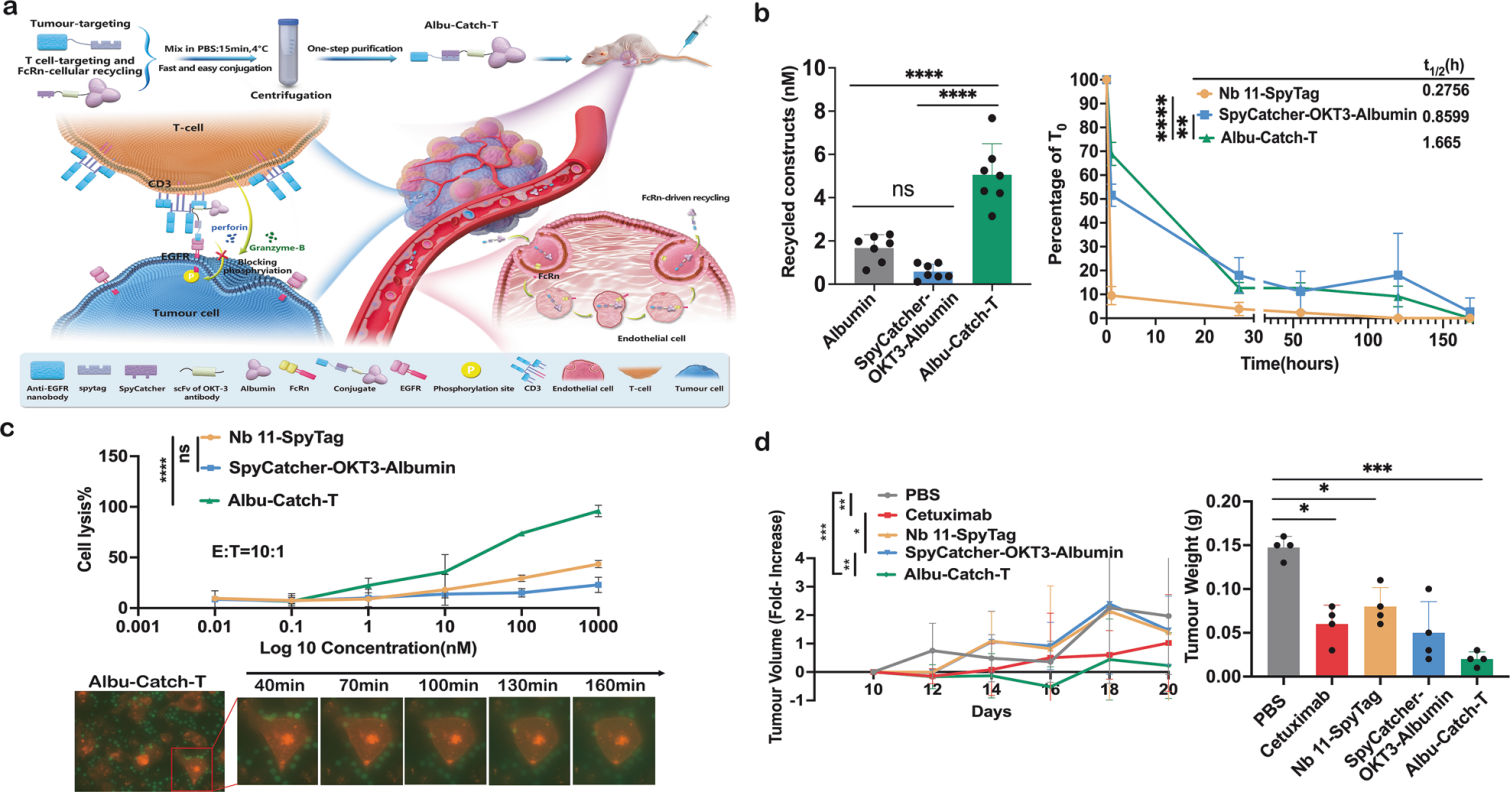
FcRn-driven half-life extension and T-cell engager anti-tumour effects of the modular *"Catch and Play"* platform. **a** Schematic representation of the *“Catch-and-Play”* modular platform design and the therapeutic mechanism of action. **b** FcRn-driven cellular recycling of recombinant human albumin, SpyCatcher-OKT3-Albumin and Albu-Catch-T in a FcRn-expressing dermal human microvascular endothelium cell line (HMEC-1-FcRn) and the pharmacokinetics of Nb 11-SpyTag, SpyCatcher-OKT3-Albumin, and Albu-Catch-T in BALB/c mice (*N* = 6). The data is shown as mean ± SD. A one-way ANOVA with multiple comparisons and Turkey’s post hoc correction was used to investigate statistical differences between the constructs ****p < 0.0001, ns-no significance. **c** In vitro cancer cell lysis induction on addition of either Nb 11-SpyTag, SpyCatcher-OKT3-Albumin or Albu-Catch-T with human PBMCs mixed with MDA-MB-231 cells at a E:T ratio of 10:1. A one-way ANOVA with multiple comparisons and Turkey’s post hoc correction was used to investigate statistical differences between the constructs ****p < 0.0001, ns-no significance. Microscopic time-lapse imaging of Albu-Catch-T activated T-cell-mediated killing of target MDA-MB-231 cells (DiI (Red) labelled) by human PBMCs (CFSE (Green) labelled). **d** In vivo efficacy in a humanised breast cancer orthotopic BALB/c nude mouse model. At day 10, either PBS, Cetuximab, Nb 11-SpyTag, SpyCatcher-OKT3-Albumin, or Albu-Catch-T mixed with PBMCs were injected (200 µL volume containing 666.67 mole protein) into the tail veins every 3 days for a total of 6 doses (Albu-Catch-T, Cetuximab, Nb 11-SpyTag, and SpyCatcher-OKT3-Albumin groups, *N* = 6; PBS group, *N* = 4). Tumour volumes were recorded every 3 days from day 10 and normalised to day 10 to show tumour volume fold-increase. At day 20 isolated tumour tissue was weighed. Generalised linear mixed models were used to investigate statistical differences between the constructs, ****p < 0.0001, ***p < 0.001, **p < 0.01, *p < 0.1, ns-no significance

SpyCatcher-OKT3-Albumin comprising of a CD3-targeting OKT3 scFv fragment and FcRn-targeting human serum albumin genetic fusion, was used for *“catching”* an anti-EGFR nanobody Nb 11previously described^3^ genetically fused to a SpyTag in the C-terminal (referred to as Nb 11-SpyTag). SpyCatcher-OKT3-Albumin expressed in HEK293E cells, and Nb-SpyTag expressed in *E.coli* were purified by anti-albumin affinity chromatography and Ni-NTA affinity chromatography, respectively. The size measured by atomic force microscopy of Nb 11-SpyTag and SpyCatcher-OKT3-Albumin was 1.9 ± 0.5 nm and 6.7 ± 1.3 nm, respectively. Whilst the binding affinity of Nb 11-SpyTag with EGFR and SpyCatcher-OKT3-Albumin with FcRn was 69.3 nM and 599 nM, respectively. The *“Catch-and-Play”* platform allowed simple assembly of the full construct by simple mixing of the Nb 11-SpyTag and SpyCatcher-OKT3-Albumin modules at 4 °C in PBS buffer for ~ 15 min by formation of an isopeptide bond,^4^ with excessive Nb 11-SpyTag driving the reaction to ~ 100%. The purity of the full construct termed Albu-Catch-T was confirmed and found to increase the size to 6.9 ± 0.8 nm. Correlation between FcRn-cellular recycling and in vivo half-life of an alternative albumin-T-cell engager fusion has been previously reported by our group.^5^ In this work, Albu-Catch-T showed higher FcRn-cellular recycling than free recombinant human albumin and SpyCatcher-OKT3-Albumin, which could reflect a molecular weight and EGFR engagement effect on cellular internalisation, which translated to a 6.04-fold and 1.93-fold increase in vivo half-life in BALB/c mice compared to non-conjugated Nb 11-SpyTag and SpyCatcher-OKT3-Albumin, respectively (Fig. 1b). A dose-dependent killing effect of Albu-Catch-T was observed by cell lysis in triple negative EGFR-expressing MDA-MB-231 breast cancer cells (Fig. 1c). Microscopic time-lapse imaging revealed a cancer cell surrounded by ~ 10 human peripheral blood mononuclear cells (PBMCs) to initiate apoptosis after 100 min at a 10:1 effector cell: target cell ratio (Fig. 1c).

A humanised breast cancer orthotopic mouse model comprising MDA-MB-231 cells, mixed with human PBMCs to ensurean immunocompetent tumour microenvironment, was used to determine in vivo efficacy of Albu-Catch-T compared to Cetuximab in female BALB/c nude mice (Fig. 1d). The same molar amount of protein mixed with PBMCs was injected into the tail vein every 3 days for a total of 6 administrations. A greater reduction in tumour volume fold-increase and weight was exhibited by Albu-Catch-T. The biocompatibility of Albu-Catch-T was supported by a maintained mouse body weight and pathology in major organs. Furthermore, physiological levels of Alanine Aminotransferase (ALT), Aspartate Aminotransferase (AST), urea, and mouse serum albumin were observed, indicative of healthy liver and kidney function (unshown data).

A novel anti-EGFR Nb 11 clone was selected as the tumour-targeting motif to introduce this *“Catch-and-Play”* modular platform. The modularity of this platform was further exemplified using an alternative EGFR-targeting clone Nb 7D12 by preparing Albu-Catch-T (Nb 7D12) and verifying its dose-dependent in vitro cytotoxicity in MDA-MB-231 cells (refer to Supplementary Materials). Future work will focus on the incorporation of alternative tumour targets. In summary, this modular platform offers potential as a rapid screening tool to accelerate clinical translation of T-cell engagers towards personalised medicine.

## Supplementary information

Supplementary Materials

## Data Availability

The authors declare that all the data supporting the findings of this study are available within the paper. All the unshown data is shared in 10.6084/m9.figshare.30854948.
