# Supplementary material for: A modular “Catch-and-Play” platform for rapid T-cell engager target assembly for personalised cancer treatment
Source: Signal Transduct Target Ther. 2026 Jan 12;11:18. doi: 10.1038/s41392-025-02557-5 (PMC12796408; doi:10.1038/s41392-025-02557-5)
Supplement: Supplementary file 1 — Supplementary Materials [file 41392_2025_2557_MOESM1_ESM.docx]

Supplementary Materials

A modular “*Catch-and-Play*” platform for rapid T-cell engager target assembly for personalised cancer treatment

Xi Xi^1,2,3^, Yonghui Zhang^2^, Daqing Zhao^3^, Fangfang Chen^1^✉️ and Kenneth A Howard ^2^✉️

✉️Correspondence to: kenh@inano.au.dk or cff@jlu.edu.cn

**This PDF file includes:**

Materials and Methods

Supplementary Text

Materials and Methods

***Protein Expression and Purification***

The sequence of Nb 11 was taken from our previous study [1]. Full-length cDNA of Nb 11-SpyTag, was inserted into vector pCold II at clone sites (5’,3’) *Ndel* and *Xbal*, and transformed into E. coli BL21 (DE3) competent cells (New England Biolabs, #C2527H) by heat shock (42ºC, 60s). One anti-ampicillin (Beyotime, #ST008) clone was screened and inoculated into 1L LB medium and cultured at 120 rpm, 37ºC to 0.6-0.8 OD value at 600nm in a shaker (ZHICHEN®-2102C). 1 mM isopropyl-β-Dthiogalactoside (IPTG) (Sigma-Aldrich, #I6758) was added to induce protein expression at 120 rpm, 16ºC overnight. After harvesting and lysing *E. coli* cells by ultra-sonification (250 W, work 10 s, off 10 s, 10 min), the supernatant was harvested by centrifugation at 9000×g, 4ºC for 30 min and the nanobodies purified using a Histrap™ HP column (1ml). Full-length cDNA of SpyCatcher-OKT3-Albumin was inserted to vector pCDNA3.1 and mammalian HEK293E cells (ATCC, Hek c18) under standard conditions by transfection grade linear PEI 40KDa; 37 °C, 5% CO₂ in serum-free Freestyle^TM^293 expression media (Gibco, #12338018). Protein was harvested from the supernatant and purified using a CaptureSelect human albumin affinity matrix (Thermo Fisher, cat#191297005). The products were analysed by using SDS–PAGE electrophoresis and Western Blot.

***Albu-Catch-T Conjugation and Purification***

The molar concentration of Spycatcher-OKT3-Albumin and Nb 11-SpyTag was diluted to 1µM and 2µM, respectively. The final molar concentration ratio of Spycatcher-OKT3-Albumin and Nb-SpyTag (Albu-Catch-T) were 1:2, 1:5 or 1:10 in PBS at 4°C, with, 1:2 selected for future experiments. Albu-Catch-T was purified by a 50kDa VivaSpin2 centrifugal concentrators (Sartorius, #VS0231). The accurate concentration of the purified proteins was measured by a Nanodrop 2000 spectrophotometer (Thermo Fisher® Scientific).

***Binding Affinity Measurement by Bio-Layer Interferometry***

Binding kinetics of Nb 11-SpyTag to recombinant Human EGFR (Raybiotech, #P00533) and SpyCatcher-OKT3-Albumin to Human FcRn (Immunitrack, #ITF02) was determined by Bio-Layer Interferometry (BLI) using an Octet Red 96e system (Sartorius). For Nb 11-SpyTag, EGFR was immobilized on Octet® Amine Reactive Second Generation (AR2G) Biosensors at a concentration of 10 nM in 10 mM acetate pH4 buffer. For binding kinetics measurements, Nb 11-SpyTag was prepared in a 5-step two-fold dilution series starting at 12.5 nM in PBS, pH7.4. For SpyCatcher-OKT3-Albumin, FcRn was immobilised on streptavidin-coated biosensors (ForteBio/Molecular Devices) at a concentration of 8 nM in PBS pH 7.4 supplemented with 0.01% Tween-20. For binding kinetics measurements, SpyCatcher-OKT3-Albumin was prepared in a 5-step two-fold dilution series starting at 3 µM in an assay buffer (25 mM Na-acetate, 25 mM NaH_2_PO_4_, 150 mM NaCl and 0.01% Tween-20 at pH 5.5). Measurements were performed at 30°C and 1000 rpm shaking with a 600s association and 900s dissociation step. Data analysis was performed using the Octet data analysis software (version 10.0.1.6) using a 1:1 interaction model curve fitting.

***FcRn Cellular Recycling Assay***

The FcRn cellular recycling assay was performed according to our previous protocol [2]. Prior to experimental use, HMEC-1-FcRn cells were washed twice in pre-warmed PBS followed by the addition of 0.15 µM SpyCatcher-OKT3-Albumin, Albu-Catch-T construct or recombinant human albumin (rHA) (300 µl per well) in Hanks balanced salt solution pre-adjusted to pH 6.0 by 1 M MES buffer. After 1 h incubation at 37 °C, 5% CO_2_, the cells were then washed 5 times in ice-cold PBS. 160 µl per well complete medium without FBS at physiological pH was then added and cells left at 37 °C, 5% CO_2_ for 1 h to allow release of internalised protein. Supernatants were collected and analysed by sandwich ELISA.

***In Vivo Pharmacokinetic Study***

Six female BALB/c mice per group were injected i.v. through the tail vein with either Nb 11-SpyTag labelled with Cy5.5, SpyCatcher-OKT3-Albumin or Albu-Catch-T at 5mg/kg. Blood samples were collected from the periocular veins in 20 µl heparinised micro capillary tubes (Marienfeld, #2911110) at 0 min, 0.5 h, 1 h, 6 h, 27 h, 55 h, 96 h, 120 h, 164 h and serum fractions were measured for Nb 11-SpyTag by fluorescence intensity, and SpyCatcher-OKT3-Albumin or Albu-Catch-T by human albumin determination using ELISA (BYabscience, #BY-EH1200444).

***Microscopic Time-lapse Live-imaging***

PBMCs (1×10^5/^well) labelled with CFSE were added to DiI-labelled MDA-MB-231 cells (seeded at 1×10^4^ /well) in RPMI-1640 Glutamax medium (Gibco, #32404-014). After co-incubation with 1000nM Albu-Catch-T at 37 °C, 5% CO2 for 30 min, the cells were continually imaged and recorded by EVOS M7000 Imaging System (Thermo Fisher®) with RFP channel (Ex/Em 531/593nm) and GFP channel (Ex/Em 470nm/525nm) for 3h. Images were recorded automatically every 30 min.

***Lactate Dehydrogenase (LDH) Cellular Toxicity Assay***

MDA-MB-231 cells (1×10^4^ /well) were seeded overnight in Nunc™ 96-well plates (Nunc, #161093). Nb 11-SpyTag, SpyCatcher-OKT3-Albumin or Albu-Catch-T was diluted in a 6-step 10-fold dilution series starting at 0.01nM in RPMI-1640 Glutamax medium (Gibco, #32404-014) and added into appropriate wells. Meanwhile, freshly isolated effector cells PBMC were added (at an E:T = 10:1) into appropriate wells. After 48 h of co-culture, cell lysate supernatants were collected for cytotoxicity analysis using the LDH cytotoxicity detection kit (Takarabio, #MK401) following the manufacturer’s protocol. Secreted human perforin and granzyme B was determined using a Human Perforin 1(PF1) ELISA Kit (BYabscience, #BY-EH117435) and human granzyme B(Gzms-B) ELISA Kit (BYabscience, #BY-EH110916) according to the manufacturers protocol.

***In Vivo Tumor Growth Inhibition***

Six female BALB/c nude mice were allocated to each of the groups, PBS, Cetuximab, Nb 11-SpyTag, SpyCatcher-OKT3-Albumin or Albu-Catch-T, respectively. Mice were inoculated in the right upper breast under anaesthesia with a subcutaneous injection of 2×10^6^ MDA-MB-231 cells mixed with 1×10^6^ freshly isolated human PBMCs in LDEV-free extracellular matrix protein Geltrex (Corning™, #356234). From Day 10, one single dose (666.67mole) of protein with

1 million PBMCs were injected into the tail veins every 3 days for a total of 6 doses. The tumours were measured every 3 days and tumour volume calculated based on the formula, Volume = π/(6 × L × W^2^). Tumour tissue were isolated and weight measured at termination of the study. The start tumour volume was shown as V_day10,_ the calculation formula used was, Tumour Volume (Fold Increase) = (V_dayX-_V_day10_)/V_day10_. Blood samples were taken following final treatment and analysed by Auto Chemistry Analyzer (Mindray Animal Care, BS-240Vet) using Aspartate Aminotransferase (AST) Kit (Mindray Animal Care, #Vet-02), Alanine Aminotransferase (ALT) Kit (Mindray Animal Care, #Vet-01), Urea Kit (Mindray Animal Care, #Vet-05)

***Supplementary Text***

The modularity of this *“Catch-and-Play”* platform was exemplified using an alternative previously published EGFR-targeting clone Nb 7D12 [3]. Both anti-EGFR clones were prepared with the same methods and the Kd value of Nb 7D12-SpyTag for EGFR was 45.73nM. *In vitro* cytotoxicity of Albu-Catch-T (containing Nb11) and Albu-Catch-T (containing Nb 7D12) was performed on MDA-MB-231 cells (target cells) at an effector (PBMC) to target cell (E:T) ratio of 10:1. As the dose increased, the cell lysis rate of Albu-Catch-T (Nb 11) increased from 9.06% (0.1nM) to 95.88% (1µM), while Albu-Catch-T (Nb 7D12) was 4.17% (0.1nM) to 67.70% (1µM). The highest cell lysis rate of Nb 11-SpyTag and Nb 7D12-SpyTag was 43.49% and 26.12%, respectively. These results indicate that “*Catch-and-Play*” modular platform can be potentially used for the design of different BiTE formats. Nb 11-SpyTag exhibited a greater inhibitory effect on MDA-MB-231 cells and greater inhibition of downstream phosphorylation than Nb 7D12, thus, providing the rationale for taking forward the Nb 11 Albu-Catch-T design for *in vivo* investigations in this work.

1. Xi, X., et al., *Identification of a novel anti-EGFR nanobody by phage display and its distinct paratope and epitope via homology modeling and molecular docking.* Mol Immunol, 128,165-174(2020).

2. Schmidt, E.G.W., et al., *Direct demonstration of a neonatal Fc receptor (FcRn)-driven endosomal sorting pathway for cellular recycling of albumin.* J Biol Chem, 292(32): 13312-13322(2017).

3. Schmitz, K.R., et al., *Structural evaluation of EGFR inhibition mechanisms for nanobodies/VHH domains.* Structure, 21(7), 1214-24(2013).
